# How does bridging social capital relate to health-behavior, overweight and obesity among low and high educated groups? A cross-sectional analysis of GLOBE-2014

**DOI:** 10.1186/s12889-019-8007-3

**Published:** 2019-12-04

**Authors:** Carlijn B. M. Kamphuis, Joost Oude Groeniger, Maartje P. Poelman, Mariëlle A. Beenackers, Frank J. van Lenthe

**Affiliations:** 1 Department of Public Health, Erasmus University Medical Centre, Rotterdam, The Netherlands; 2 Department of Interdisciplinary Sciences, Utrecht University, PO Box 80140, 3508 TC Utrecht, The Netherlands; 3 Department of Human Geography and Spatial Planning, Utrecht University, Utrecht, The Netherlands

**Keywords:** Bridging social capital, Socioeconomic position, Educational level, Health inequalities, Network heterogeneity, Diet, Physical activity, Overweight, Obesity

## Abstract

**Background:**

Social capital is an important determinant of health, but how specific sub-dimensions of social capital affect health and health-related behaviors is still unknown. To better understand its role for health inequalities, it is important to distinguish between bonding social capital (connections between homogenous network members; e.g. similar educational level) and bridging social capital (connections between heterogeneous network members). In this study, we test the hypotheses that, 1) among low educational groups, bridging social capital is positively associated with health-behavior, and negatively associated with overweight and obesity, and 2) among high educational groups, bridging social capital is negatively associated with health-behavior, and positively with overweight and obesity.

**Methods:**

Cross-sectional data on educational level, health-behavior, overweight and obesity from participants (25–75 years; Eindhoven, the Netherlands) of the 2014-survey of the GLOBE study were used (*N* = 2702). Social capital (“How many of your close friends have the same educational level as you have?”) was dichotomized as: bridging (‘about half’, ‘some’, or ‘none of my friends’), or bonding (‘all’ or ‘most of my friends’). Logistic regression models were used to study whether bridging social capital was related to health-related behaviors (e.g. smoking, food intake, physical activity), overweight and obesity, and whether these associations differed between low and high educational groups.

**Results:**

Among low educated, having bridging social capital (i.e. friends with a higher educational level) reduced the likelihood to report overweight (OR 0.73, 95% CI 0.52–1.03) and obesity (OR 0.58, 95% CI 0.38–0.88), compared to low educated with bonding social capital. In contrast, among high educated, having bridging social capital (i.e. friends with a lower educational level) increased the likelihood to report daily smoking (OR 2.11, 95% CI 1.37–3.27), no leisure time cycling (OR 1.55, 95% CI 1.17–2.04), not meeting recommendations for vegetable intake (OR 2.09, 95% CI 1.50–2.91), and high meat intake (OR 1.39, 95% CI 1.05–1.83), compared to high educated with bonding social capital.

**Conclusions:**

Bridging social capital had differential relations with health-behavior among low and high educational groups. Policies aimed at reducing segregation between educational groups may reduce inequalities in overweight, obesity and unhealthy behaviors.

## Background

Social capital is acknowledged as an important ‘social determinant of health’ that can promote (or harm) health through several mechanisms [1–4]. Two broad streams of research have emerged with regard to social capital. The first conceptualizes social capital on the collective level, as the resources available to members of a community such as trust, or exercise of sanctions; well-known from the work of Putnam [5]. The second stream conceptualizes social capital on the individual level, i.e. as the resources that are embedded within an individual’s social network, e.g. social support, norms; as in the work of Bourdieu [6, 7]. Both collective and individual social capital are independently associated with health [8–10], but via different pathways. In this study, we focus on individual-level social capital (hereafter: social capital, unless indicated otherwise). Although there is an abundance of evidence that confirms relationships between socioeconomic position, social capital and health in general, fewer studies have tested more specific underlying pathways, e.g. the more detailed roles of sub-dimensions of social capital, or differential roles of social capital for low and high socioeconomic groups [1, 3, 7–11].

In relation to health inequalities, the distinction between bonding and bridging social capital is particularly important. Bonding social capital refers to “inward-looking” connections between members of a network who are similar to each other (for instance with respect to ethnicity, age, or social class), which enhances access to internal resources, possibly by reinforcing exclusive social identities [2, 12, 13]. Bridging social capital, by contrast, refers to the “outward-looking” connections between members of a network who are *dissimilar* to each other, and thus to ties between heterogeneous, socially diverse groups which may enhance access to external resources [2, 12, 13]. Interactions that represent bridging prospects have declined over time, which has been observed in the U.S. [14] as well as other Western societies [15, 16], like the Netherlands. In the Netherlands, educational attainment is the most important dimension of segregation, and it has been shown that low and high educated people increasingly live separate lives, with different preferences and different lifestyles [15, 17]. Also, health-related behaviors differ remarkably when comparing low and high educated groups – more so than when comparing income or occupational groups [18–20]. Therefore, in the Dutch context, it is especially relevant to understand how *education-specific* bridging social capital (i.e. having friends with a higher or lower educational level compared to one’s own educational level) relates to health-behavior.

We hypothesize that low educated groups with education-specific *bridging* social capital, i.e. ties to higher educated groups, may be more likely to behave healthily, than low educated with bonding social capital. We reason that high educated, more often than low educated, behave healthily, have positive attitudes towards health-behavior, and provide social support for health-behavior [21–24], and that therefore, low educated with higher educated friends (i.e. bridging social capital) may be more likely to experience descriptive norms and social support for health-behavior, or to (unconsciously) mimic their higher-educated friends’ health-behavior. For high educated groups, the reverse may be true: high educated with bridging social capital (i.e. friends with a lower educational level) may be more likely to behave unhealthily, compared to high educated with bonding social capital. These hypotheses have not been tested before. Therefore, this study addresses the following research question: to what extent is bridging social capital differentially related to health-behavior of high and low educated groups?

## Methods

Data were collected by means of a large-scale postal survey in 2014, administered as the fifth wave of data collection for the longitudinal Dutch GLOBE study (response = 45.5%) [18]. A cross-sectional stratified sample of the 25–75 years old population in the city of Eindhoven was used in the analyses (*N* = 2812) [20, 25]. More detailed information on the objectives, study design, and data collection of the Dutch GLOBE study can be found elsewhere [26, 27]. The use of personal data in the GLOBE study is in compliance with the Dutch Personal Data Protection Act and the Municipal Database Act, and has been registered with the Dutch Data Protection Authority (number 1248943).

### Educational level and education-specific bridging social capital

Educational level is an important indicator of social stratification in contemporary Dutch society [15, 17, 28]. Survey participants reported their highest attained educational level, which was classified according to the International Standard Classification of Education (ISCED): 1– high education (tertiary education (ISCED 5–7)); 2– mid education (upper secondary education (ISCED 3–4)); 3– low education (primary education and lower secondary education (ISCED 0–2)).

Education-specific bridging social capital was measured with the question: “How many of your close friends have the same educational level as you have?”, with five answering options: all, most of them, about half, some, and none. Bridging social capital was coded as ‘1 = bridging’ for those who answered ‘about half’, ‘some’, or ‘none of my friends’, and ‘0 = bonding’ for those who answered ‘all’ or ‘most of my friends’.

### Health-behavior, overweight and obesity

Sports participation was measured by means of the validated Short QUestionnaire to ASsess Health-enhancing physical activity (SQUASH) [29]. Participants were asked to think about their sports participation over the past month and to write down up to four different types of sports they had participated in on a weekly basis. For each type of sport they reported frequency (days per week), duration (hours and minutes per day) and intensity (light, moderate, intense). Self-reported intensity and activity-specific intensity Metabolic Equivalents (METs) were used to calculate the numbers of days participants participated in sports for at least 30 min at moderate or vigorous intensity (moderate intensity = 4–6 MET for 18–55 years and 3–5 MET for 55+ years). The variable was dichotomized into 1) no sports participation at least once per week for > 30 min at moderate intensity, vs. 0) sports participation at least once per week for > 30 min at moderate intensity (reference group).

Walking and cycling in leisure time were also measured as part of the SQUASH. Participants reported frequency (days per week), duration (hours and minutes per day) and intensity (light, moderate, intense) for walking and cycling in their leisure time. For both activities separately, we calculated how many days per week the participant walked and cycled for at least 30 min at moderate intensity. Both variables were dichotomized: 1) no walking at least once per week for > 30 min at moderate intensity, vs. 0) walking at least once per week for > 30 min at moderate intensity (reference group); and 1) no cycling at least once per week for > 30 min at moderate intensity, vs. 0) cycling at least once per week for > 30 min at moderate intensity (reference group).

Fruit and vegetable consumption were measured as part of a food frequency questionnaire [30]. Participants reported frequency (days per week) over the past month, for fruit and vegetable consumption separately. They also indicated the portion size (i.e. number of pieces of fruit, and number of serving spoons [=50 g] of vegetables) on a typical occasion. Based on this, we calculated whether participants did or did not meet recommendations for fruit and vegetable consumption. Those who did not consume two pieces of fruit every day were coded as “not meeting recommended fruit intakes” (and those eating two or more pieces of fruit every day were used as reference group). Those who not consumed 200 g of vegetables every day were coded as “not meeting recommended vegetable intakes” (with eating > 200 g of vegetables every day as reference group).

Water intake was also part of the food frequency questionnaire. Participants reported how many days per week, over the past month, they consumed tap water or spring water. Water consumption less than daily was coded as ‘no daily water intake’ (with ‘daily water intake’ as reference group). Further, participants reported how many days per week, over the past month, they consumed meat. Meat intake on 5 till 7 days per week was coded as ‘high meat intake’, whereas meat intake on less than 5 days per week was coded ‘no high meat intake’ (reference group).

Body mass index (BMI) was calculated by self-reported height and weight. Participants with a BMI higher than 25 were categorized as overweight (and BMI < 25 as no overweight, reference group). A BMI higher than 30 was categorized as obesity (and BMI < 30 as no obesity, reference group). Overweight and obesity were applied as outcomes since these are related to two types of health-behaviour: diet and physical activity.

### Confounders

Potential confounders were included in all analyses: sex (male, female), age (in 10-year age groups), country of birth (Netherlands, other), living together with a partner (yes, no), children living in your household (yes, no), employment status (employed, unemployed, retired, or other (e.g. homemaker, student)), father’s highest attained educational level, and mother’s highest attained educational level. The latter two were included as confounders, since parental education level could influence the likelihood that low educated become friends with higher educated, *and* the likelihood to behave healthily (and the opposite for high educated becoming friends with lower educated). Low educated who were raised by high-educated parents may be more likely to get in touch with higher educated (e.g. directly, via their parents connections, or indirectly, as they have learned to speak the ‘appropriate’ language to connect with higher educated [31]). Also, low educated with high educated parents may be raised in a sociocultural environment in which healthy behavior was more prevalent.

### Statistical analysis

Participants with missing values for educational level or the confounders sex, age, country of birth, living with a partner, children, or employment status, were excluded from the analyses (*n* = 110). This resulted in an analytic sample of *N* = 2702. To estimate the main effects of bridging social capital and educational level on the outcomes, a logistic regression model was run for each outcome separately, including bridging social capital, educational level, and confounders (results in Additional file 1). To examine whether the association between bridging social capital and health-behavior was modified by educational level, Knol and VanderWeele’s template for reporting analysis investigating effect modifications was used [32]. Their recommendations include running three different logistic regression models for each outcome (referred to as model A, B and C below), in order to provide readers with sufficient information to draw conclusions on the size and statistical significance of the effect modification [32]. Model A is most commonly used in the field of public health for calculating effect modification (or interaction), namely a model with educational level, bridging social capital and confounders, plus a multiplicative interaction term between educational level and bridging social capital. The results from model A are presented in the footnotes of Tables 2-4, and used as an overall test of whether education significantly modifies the association between bridging social capital and outcomes. In model B, odds ratios (ORs) for each combination of educational level and bridging capital were calculated. Thereto, we first composed a combined ‘education*capital’-variable, resulting in a variable with six categories (1 = high education with bonding capital (reference group); 2 = high education with bridging capital; 3 = mid-education with bonding capital; 4 = mid education with bridging capital; 5 = low education with bonding capital; 6 = low education with bridging capital). In model B, this combined education*capital-variable and confounders were included. In model C, bridging social capital and confounders were included in a regression model, and results were stratified by educational level. In this way, model C produced ORs for the association between bridging social capital and the outcomes for each educational group separately (low, mid and high educated). All statistical analyses were conducted in SPSS 23.0.

## Results

Bridging social capital was more prevalent among low educated (47.7%) than high educated (22.3%). Low educated were older, more often retired or unemployed, and more often lived without children in their household (Table 1). Overall, bridging social capital increased the likelihood of daily smoking, no sports participation, no leisure time cycling, and not meeting recommendations for vegetable intake, compared to bonding social capital (see Additional file 1). Also, educational inequalities in health-behavior were observed, with low educated and mid-educated more likely to report unhealthy behavior, overweight and obesity compared to high educated (see Additional file 1).

**Table 1:** Study sample characteristics of the GLOBE-2014 sample

		Educational level*		
	Total	1-High	2-Mid	3 -Low
	(*N* = 2702)	(*n* = 1292)	(*n* = 673)	(*n* = 737)
	%	%	%	%
Gender				
Man	44.6	48.5	44.9	36.9
Woman	55.4	51.5	55.1	63.1
Age groups				
25–34 years	25.7	36.3	23.5	7.2
35–44 years	17.1	19.6	21.1	8.4
45–54 years	17.6	15.6	24.2	15.3
55–64 years	19.3	16.2	17.1	27.6
65–75 years	20.2	12.4	14.2	41.5
Living together with a partner				
Yes	74.1	75.4	70.8	75.0
No	25.9	24.6	29.2	25.0
Country of birth				
Netherlands	89.0	89.9	89.0	87.3
Else	11.0	10.1	11.0	12.7
Children living at home				
No	64.2	61.8	57.2	76.0
Yes	35.8	38.2	42.8	24.0
Employment status				
Employed	63.9	77.0	68.4	33.7
Unemployed	7.9	4.9	8.2	13.5
Retired	20.4	13.3	15.0	39.6
Homemaker, student, other	7.9	4.8	8.3	13.3
Father’s educational level				
High	23.9	39.3	14.4	3.2
Mid	18.8	22.6	23.0	7.3
Low	38.2	27.1	42.7	55.4
Missing	19.1	11.0	19.8	34.1
Mother’s educational level				
High	12.4	21.5	5.7	1.2
Mid	17.5	25.1	17.4	2.9
Low	52.2	43.7	57.9	63.0
Missing	17.9	9.7	19.1	32.8
*Bridging/bonding social capital*				
Bridging social capital	33.6	22.3	43.3	47.7
Bonding social capital	66.4	77.7	56.7	52.3
*Health-behavioral outcomes*				
Daily smoking	15.4	7.8	20.8	25.1
No sports participation	37.5	26.6	40.4	56.1
No walking in leisure time	66.2	72.8	72.6	46.3
No cycling in leisure time	41.9	41.8	44.2	39.8
No recommended veg. intake	76.6	71.1	82.5	81.9
No recommended fruit intake	66.3	64.2	72.2	64.7
No daily water intake	23.1	19.6	26.8	26.2
High meat intake	57.4	55.2	59.6	59.6
Overweight	46.9	35.8	53.0	62.5
Obesity	13.3	8.0	16.2	20.9

*Chi-square tests indicated that educational differences in the reported characteristics were significant (*p* < .001), with the exception of country of birth (p: 0.209), living with a partner (p: .062), no cycling in leisure time (p: .277), and high meat intake (p: .061)

The results of model A indicated that educational level significantly modified the relation between bridging social capital and outcomes, except for sports participation, leisure time walking and fruit intake (see the *p*-values in the footnotes of Tables 2-4). The results of models B and C give more insight in the direction and size of the effect modification. Regarding health-behavior (Tables 2-3), bridging social capital mattered mostly for high educated. Among high educated, having bridging social capital increased the likelihood to report daily smoking (OR 2.11, 95% CI 1.37–3.27), no leisure time cycling (OR 1.55, 95% CI 1.17–2.04), not meeting recommendations for vegetable intake (OR 2.09, 95% CI 1.50–2.91), and a high meat intake (OR 1.39, 95% CI 1.05–1.83). On the other hand, regarding overweight and obesity (Table 4), bridging social capital mattered mostly for low educated, but in the opposite direction than among high educated. Among low educated, bridging social capital *reduced* the likelihood to report overweight (OR 0.73, 95% CI 0.52–1.03) (non-significant) and obesity (OR 0.58, 95% CI 0.38–0.88).

**Table 2:** Multivariable logistic regression models for modification of the association between bridging social capital^a^ and daily smoking, no sports participation, no leisure time walking, and no leisure time cycling, by educational level

	Bonding social capital^a^			Bridging social capital^a^			ORs (95% CI) for bridging capital within educational groups	
Daily smoking								
Education	N smoking vs. N non-smoking	OR ^b^	(95% CI)	N smoking vs. N non-smoking	OR^b^	(95% CI)	OR^c^	(95% CI)
High	62 vs. 897	1.00		33 vs. 252	2.08***	**(1.35–3.19)**	2.11***	**(1.37–3.27)**
Medium	69 vs. 288	3.75***	**(2.59–5.42)**	54 vs. 219	3.50***	**(2.35–5.22)**	1.01	(0.67–1.51)
Low	69 vs. 258	5.14***	**(3.37–7.84)**	85 vs. 207	7.34***	**(4.87–11.07)**	1.35	(0.90–2.03)
No sports participation								
Education	N no sports vs. N doing sports	OR ^b^	(95% CI)	N no sports vs. N doing sports	OR^b^	(95% CI)	OR^c^	(95% CI)
High	218 vs. 674	1.00		82 vs. 182	1.29	(0.96–1.75)	1.32	(0.97–1.80)
Medium	122 vs. 213	1.43**	**(1.09–1.87)**	106 vs. 140	1.94***	**(1.44–2.61)**	1.37	(0.97–1.94)
Low	166 vs. 142	2.27***	**(1.68–3.08)**	153 vs. 121	2.52***	**(1.85–3.44)**	1.12	(0.80–1.58)
No leisure time walking								
Education	N no walking vs. N walking	OR^b^	(95% CI)	N no walking vs. N walking	OR^b^	(95% CI)	OR^c^	(95% CI)
High	695 vs. 220	1.00		199 vs. 67	1.04	(0.66–1.63)	1.01	(0.63–1.59)
Medium	243 vs. 97	1.28	(0.84–1.94)	185 vs. 72	1.01	(0.62–1.64)	0.80	(0.43–1.48)
Low	131 vs. 173	1.20	(0.78–1.85)	134 vs. 124	1.85**	(1.19–2.87)	1.49	(0.94–2.37)
No leisure time cycling								
Education	N no cycling vs. N cycling	OR^b^	(95% CI)	N no cycling vs. N cycling	OR^b^	(95% CI)	OR^c^	(95% CI)
High	365 vs. 535	1.00		124 vs. 138	1.58***	**(1.20–2.08)**	1.55**	**(1.17–2.04)**
Medium	157 vs. 176	1.36*	**(1.05–1.76)**	113 vs. 143	1.22	(0.91–1.63)	0.95	(0.68–1.34)
Low	115 vs. 174	1.48*	**(1.08–2.02)**	109 vs. 141	1.62**	**(1.18–2.24)**	1.12	(0.77–1.63)

* = *p* < .050, ** = *p* < .010, *** = *p* < .001

^a^ Bridging social capital was measured with the question: “How many of your close friends have the same educational level as you have?”. Bridging social capital was coded as ‘1 = bridging’ for those who answered ‘about half’, ‘some’, or ‘none of my friends’, and ‘0 = bonding’ for those who answered ‘all’ or ‘most of my friends’

^b^ Odds ratios and 95% confidence intervals regressing the outcomes on the ‘education x capital’-variable and confounders (sex, age, employment status, country of birth, living with partner, children in household, father’s education, mother’s education)

^c^ Odds ratios and 95% confidence intervals regressing the outcomes on bridging social capital and confounders, stratified by educational level.- Measures on multiplicative scale regarding daily smoking: OR for education medium* bridging capital = 0.45 (95% CI 0.25–0.80), ***P*** **= 0.007**; OR for education low * bridging capital: = 0.69 (95% CI 0.39–1.22), *P* = 0.201- Measures on multiplicative scale regarding no sports participation: OR for education medium* bridging capital = 1.05 (95% CI 0.67–1.64), *P* = 0.833; OR for education low * bridging capital: = 0.86 (95% CI 0.55–1.34), *P* = 0.499.- Measures on multiplicative scale regarding no leisure time walking: OR for education medium* bridging capital = 0.77 (95% CI 0.38–1.55), *P* = 0.457; OR for education low * bridging capital: = 1.50 (95% CI 0.79–2.83), *P* = 0.216.- Measures on multiplicative scale regarding no leisure time cycling: OR for education medium* bridging capital = 0.57 (95% CI 0.37–0.87), ***P*** **= 0.009**; OR for education low * bridging capital: = 0.70 (95% CI 0.44–1.09), *P* = 0.113

**Table 3:** Multivariable logistic regression models for modification of the association of bridging social capital^a^ on vegetable intake, fruit intake, water intake, and meat intake, by educational level

	Bonding social capital^a^			Bridging social capital^a^			ORs (95% CI) for bridging capital within educational groups	
No recommended vegetable intake								
Education	N no recomm. vs. N recomm. vegetable intake	OR^b^	(95% CI)	N no recomm. vs. N recomm. vegetable intake	OR^b^	(95% CI)	OR^c^	(95% CI)
High	644 vs. 306	1.00		224 vs. 57	2.03***	**(1.47–2.82)**	2.09***	**(1.50–2.91)**
Medium	291 vs. 63	2.14***	**(1.57–2.92)**	218 vs. 51	2.12***	**(1.49–3.02)**	0.99	(0.64–1.55)
Low	269 vs. 54	2.02***	**(1.42–2.88)**	232 vs. 54	1.98***	**(1.38–2.85)**	0.96	(0.62–1.48)
No recommended fruit intake								
Education	N no recomm. vs. N recomm. fruit intake	OR^b^	(95% CI)	N no recomm. vs. N recomm. fruit intake	OR^b^	(95% CI)	OR^c^	(95% CI)
High	614 vs. 342	1.00		199 vs. 85	1.25	(0.94–1.67)	1.25	(0.94–1.67)
Medium	247 vs. 119	1.48**	**(1.13–1.95)**	191 vs. 78	1.40*	**(1.03–1.91)**	0.96	(0.66–1.38)
Low	201 vs. 122	1.38*	**(1.01–1.87)**	183 vs. 101	1.30	(0.95–1.78)	0.92	(0.64–1.31)
No daily water intake								
Education	N no daily water vs. N daily water intake	OR^b^	(95% CI)	N no daily water vs. N daily water intake	OR^b^	(95% CI)	OR^c^	(95% CI)
High	171 vs. 785	1.00		66 vs. 218	1.27	(0.93–1.74)	1.29	(0.94–1.77)
Medium	99 vs. 257	1.77***	**(1.33–2.35)**	63 vs. 211	1.23	(0.89–1.71)	0.66*	**(0.45–0.96)**
Low	80 vs. 250	1.61**	**(1.16–2.25)**	78 vs. 210	1.65**	**(1.18–2.31)**	1.08	(0.74–1.56)
High meat intake								
Education	N high meat vs. N no high meat intake	OR^b^	(95% CI)	N high meat vs. N no high meat intake	OR^b^	(95% CI)	OR^c^	(95% CI)
High	512 vs. 446	1.00		171 vs. 113	1.42*	**(1.08–1.86)** **.0)**	1.39*	**(1.05–1.83)**
Medium	226 vs. 133	1.38*	**(1.07–1.77)**	160 vs. 114	1.25	(0.94–1.66)	0.94	(0.67–1.31)
Low	210 vs. 120	1.81***	**(1.35–2.44)**	162 vs. 128	1.43*	**(1.06–1.93)**	0.81	(0.58–1.14)

* = *p* < .050, ** = *p* < .010, *** = *p* < .001

^a^ Bridging social capital was measured with the question: “How many of your close friends have the same educational level as you have?”. Bridging social capital was coded as ‘1 = bridging’ for those who answered ‘about half’, ‘some’, or ‘none of my friends’, and ‘0 = bonding’ for those who answered ‘all’ or ‘most of my friends’

^b^ Odds ratios and 95% confidence intervals regressing the outcomes on the ‘education x capital’-variable and confounders (sex, age, employment status, country of birth, living with a partner, children in household, father’s education, mother’s education)

^c^ Odds ratios and 95% confidence intervals regressing the outcomes on bridging social capital and confounders, stratified on educational level.- Measures on multiplicative scale regarding no recommended vegetable intake: OR for education medium * bridging capital = 0.49 (95% CI 0.29–0.83), ***P*** **= 0.008**; OR for education low * bridging capital: = 0.48 (95% CI 0.28–0.82), ***P*** **= 0.007**.- Measures on multiplicative scale regarding no recommended fruit intake: OR for education medium* bridging capital = 0.75 (95% CI 0.48–1.19), *P* = 0.220; OR for education low * bridging capital: = 0.75 (95% CI 0.48–1.18), *P* = 0.213.- Measures on multiplicative scale regarding no daily water intake: OR for education medium* bridging capital = 0.55 (95% CI 0.34–0.88), ***P*** **= 0.012**; OR for education low * bridging capital: = 0.80 (95% CI 0.49–1.30), *P* = 0.368.- Measures on multiplicative scale regarding high meat intake: OR for education medium* bridging capital = 0.64 (95% CI 0.42–0.98), ***P*** **= 0.038**; OR for education low * bridging capital: = 0.56 (95% CI 0.36–0.86), ***P*** **= 0.007**

**Table 4:** Multivariable logistic regression models showing ORs for modification of the effect of having bridging social capital^a^ on overweight (BMI > 25) and obesity (BMI > 30), by educational level

	Bonding social capital^a^			Bridging social capital^a^			ORs (95% CI) for bridging capital within educational groups	
Overweight								
Education	N overweight vs. N no overweight	OR^b^	(95% CI)	N overweight vs. N no overweight	OR^b^	(95% CI)	OR^c^	(95% CI)
High	355 vs. 698	1.00		123 vs. 180	1.18	(0.89–1.55)	1.15	(0.86–1.52)
Medium	201 vs. 175	1.92***	**(1.49–2.48)**	150 vs. 142	1.77***	**(1.34–2.34)**	0.87	(0.63–1.22)
Low	209 vs. 115	2.42***	**(1.80–3.24)**	165 vs. 128	1.78***	**(1.32–2.39)**	0.73	(0.52–1.03)
Obesity								
Education	N obesity vs. N no obesity	OR^b^	(95% CI)	N obesity vs. N no obesity	OR^b^	(95% CI)	OR^c^	(95% CI)
High	70 vs. 880	1.00		28 vs. 255	1.25	(0.80–1.96)	1.24	(0.78–1.96)
Medium	58 vs. 292	2.00***	**(1.38–2.90)**	46 vs. 227	2.01***	**(1.34–3.01)**	0.97	(0.63–1.51)
Low	85 vs. 242	2.65***	**(1.79–3.92)**	52 vs. 236	1.53	(0.99–2.36)	0.58*	**(0.38–0.88)**

* = *p* < .050, ** = *p* < .010, *** = *p* < .001

^a^ Bridging social capital was measured with the question: “How many of your close friends have the same educational level as you have?”. Bridging social capital was coded as ‘1 = bridging’ for those who answered ‘about half’, ‘some’, or ‘none of my friends’, and ‘0 = bonding’ for those who answered ‘all’ or ‘most of my friends’

^b^ Odds ratios and 95% confidence intervals regressing the outcomes on the ‘education x capital’-variable and confounders (sex, age, employment status, country of birth, living with a partner, children in household, father’s education, mother’s education)

^c^ Odds ratios and 95% confidence intervals regressing the outcomes on bridging social capital and confounders, stratified on educational level.- Measures on multiplicative scale regarding overweight: OR for education medium* bridging capital = 0.80 (95% CI 0.53–1.22), *P* = 0.301; OR for education low * bridging capital: = 0.65 (95% CI 0.42–0.99), ***P*** **= 0.047**.- Measures on multiplicative scale regarding obesity: OR for education medium* bridging capital = 0.81 (95% CI 0.44–1.49), *P* = 0.490; OR for education low * bridging capital: = 0.46 (95% CI 0.25–0.85), ***P*** **= 0.013**

## Discussion

### Main findings

Different relations of bridging social capital with health-behavior, overweight and obesity for low and high educated were found. This partly confirmed our hypothesis that bridging social capital would have beneficial relations with health-behavioral outcomes for low educated, but not for high educated. Indeed, among low educated, bridging social capital reduced the likelihood of overweight and obesity, but did not reduce the likelihood of unhealthy behavior. Among high educated, bridging social capital increased the likelihood of some unhealthy behaviors: smoking, low cycling levels, low vegetable intake, and high meat intake.

### Interpretation in light of the literature

Research into bridging social capital suffers from the lack of a standardized measurement approach [13], which makes it difficult to compare our results to previous studies. A recent review found evidence that collective social capital had a stronger positive association with good health for people with a lower socioeconomic status, and may buffer against the negative health effects of a low socioeconomic status [3]. This parallels our findings with respect to overweight and obesity. Two Japanese studies and a British study were somewhat similar to our study in the sense that these measured individual-level bridging social as some degree of heterogeneity in social contacts [12, 33, 34]. Where we found that bridging social capital increased the odds of some types of unhealthy behavior (but had no relations with other), these studies showed that bridging social capital had beneficial relations with several outcomes: a lower odds of physical inactivity [33], inverse associations with depressive mood [12], and positive associations with self-rated health [34]. Differences in the measurement of bridging social capital likely contribute to the contrasting results: whereas we measured bridging social capital referring to (dis) similarities in the educational level of close friends, other studies referred to more general connections (network connections, participants of community activities), and (dis) similarities in ‘social characteristics’ in general (e.g. age, sex) [12, 33] or ethnicity and income [34].

Our study extends previous research by investigating whether associations of bridging social capital with health-behavior differ for educational groups. Indeed, bridging social capital increased the likelihood of smoking, low cycling levels, low vegetable intake, and high meat intake among high educated groups, but was unrelated to health-behavior of low educated. An explanation for this may be that healthy behavior, for most people, requires more effort and constraint than unhealthy behavior (e.g. it is often easier to be inactive than sufficiently active, and it is easier to eat few than recommended amounts of vegetables). It seems as if behaving healthily is most ‘doable’ for high educated with high educated friends (i.e. with bonding social capital), possibly because they experience stronger descriptive norms, and more social support and role-modelling to adopt and maintain healthy behavior, than high educated with low educated friends. High educated with bridging social capital (i.e. with lower educated friends) may perceive less social support, or less strict descriptive norms, making them more likely to adopt unhealthy behavior.

Our results for overweight and obesity seem in contrast to this explanation: bridging social capital *did* reduce the likelihood to be overweight and obese among low educated, although one could similarly argue for overweight/obesity that the unhealthy option (putting on weight) is easier than the healthy option (weight control). However, there is an important difference between overweight/obesity and health-behavior that could play a role here: there is a strong slimness ideal in Western societies, and such a strong ideal is lacking for most types of health-behavior. Although this slimness ideal may be strongest among high educated, qualitative studies show that weight control and bodily appearance also matter to low educated [35, 36], whereas health-behavior on the other hand is more often rejected or opposed by low educated (e.g. ‘healthy food is tasteless, boring and insufficiently satisfying’) [36]. So, as interpretation of our findings for obesity, we surmise that these may show that the healthy norm outweighs the unhealthy norm. For this reason, it may be that low educated with higher educated friends have a *reduced* risk of obesity (instead of high educated with low educated friends having an *increased* risk of obesity - what we observed for health behavior).

The previous interpretations assume a causal relation between bridging capital and health-behavior. However, as the cross-sectional design of our study does not give insight in the direction of the observed relations, another plausible explanation may be that ‘like attracts like’: low educated with a healthy lifestyle may 'select' friends with a similar healthy lifestyle, who are more likely to be higher educated. Studies from sociology and social psychology show that, before all, the composition and structure of personal networks is affected by the social contexts that a person enters during daily life, such as the work place, school and voluntary associations [37]. Who then, of all people you meet within these contexts, become your friends, is further determined by similarities in age, sex, ethnicity, educational level [38], and personality [39]. The role of people’s health-behavior in the process of who becomes friends with whom, is less well known.

Since, in the Netherlands, educational inequalities in health-behavior are larger than those by income (and occupation), we expected education-specific bridging links to matter more for health-behavior than income-specific bridging links. We tested this assumption in additional analyses. In our survey, respondents indicated their income level, and how many close friends had a similar income level. In additional analyses, we tested whether income level modified the association between income-specific bridging capital and health-behavior. No meaningful effect modification was found (results in Additional file 2). Apparently, in the Netherlands, educational level is the crucial dimension of bridging social capital in relation to health-behavior inequalities. In other societies, in which race and income level are important dimensions of societal segregation, bridging social capital with respect to race and income level may be just as important.

### Strengths & limitations

An important strength of this study is that we conducted a more detailed measurement of bridging social capital compared to previous studies. Also, we tested whether associations of bridging social capital differed for educational groups. Thereby, this study took a differentiated approach to bridging capital, which has often been called for [3, 7, 13], providing more insight in possible underlying pathways between socioeconomic position, bridging social capital and health-behavior. Further, we measured multiple types of health-behavior, as well as overweight and obesity, which allowed us to rigorously test our hypothesis. Lastly, we controlled our cross-sectional analyses for a wide range of potential confounders, including parental education. In this way, we tried to rule out confounding effects as much as possible, by controlling for factors that may affected the respondents’ educational level, bridging social capital and/or health-behavior.

As said, the most important limitation of our study is its cross-sectional design, which does not give insight in the direction of the relations observed. Another limitation is that our data were collected by means of a survey, which may have led to a selective sample of respondents, i.e. those interested in, and capable of, filling in a 16-page survey on health and living conditions. We may have missed the lowest educated, and therefore, the educational inequalities in health-behavior we found are likely an underestimation of the real inequalities. Further, we excluded respondents from the analyses with missing data on one of the confounders (*n* = 110). This excluded group was lower educated, older, more often retired, not born in the Netherlands, and more often reported overweight, obesity, and no sports participation. Also for this reason, the health-behavioral inequalities we found are likely an underestimation of the true inequalities. In order to check the representativeness of our sample for the local population, we compared prevalence rates of the health-behaviors in our survey, compared to a survey carried out by the municipal health service in the same target population, which showed that prevalence rates were comparable. However, the comparison data likely suffers from similar caveats as these were also collected by means of a survey. As the lowest socioeconomic groups are likely underrepresented in both datasets, the generalization of our results to these groups is limited.

A final limitation is related to our measure of education-specific bridging capital. We did not ask whether close friends had a higher or lower educational level, but just whether they had a *different* educational level compared to respondent. This is problematic for the mid-educated, as the answer that most friends have a different educational level does not reveal whether they were lower or higher educated. Future research should consider measuring education-specific bridging social capital with two questions, asking respondents what proportion of their friends has a higher, and what proportion has a lower educational level than themselves.

## Conclusion

In contemporary societies one’s social position is increasingly determined by societal successes, including educational achievements. As a result, low and high educated citizens lead increasingly segregated lives, and few bridges between educational groups remain. The results of our study may imply that lower levels of bridging social capital in contemporary societies may lead to more obesity among low educated groups, and healthier behavior among high educated groups, which would then lead to a widening of health inequalities. However, since our study is cross-sectional, future studies should give more insight in the causal relations of bridging social capital with health-behavior, overweight and obesity among different educational groups, and the underlying mechanisms.

## Supplementary information

**Additional file 1.** Main effects of educational level and education-specific bridging social capital, adjusted for confounders

**Additional file 2.** Models for associations between income-specific bridging social capital and health-behavior, modified by income level

## Data Availability

The datasets generated during and/or analysed during the current study are not publicly available due to privacy regulations, but are available from the corresponding author on reasonable request.

## References

[1] Kawachi I, Berkman LF, Berkman LF, Kawachi I (2000). Social cohesion, social capital, and health. Social Epidemiology.

[2] Moore S, Kawachi I (2017). Twenty years of social capital and health research: a glossary. J Epidemiol Community Health.

[3] Uphoff EP, Pickett KE, Cabieses B, Small N, Wright J (2013). A systematic review of the relationships between social capital and socioeconomic inequalities in health: a contribution to understanding the psychosocial pathway of health inequalities. Int J Equity Health.

[4] Poortinga W (2006). Do health behaviors mediate the association between social capital and health?. Prev Med.

[5] Lochner K, Kawachi I, Kennedy BP (1999). Social capital: a guide to its measurement. Health Place.

[6] Bourdieu P, Richardson R (1986). The forms of capital. Handbook of theory and research for the sociology of education.

[7] Ferlander S (2007). The importance of different forms of social capital for health. Acta Sociologica.

[8] De Clercq B, Vyncke V, Hublet A, Elgar FJ, Ravens-Sieberer U, Currie C (2012). Social capital and social inequality in adolescents' health in 601 Flemish communities: a multilevel analysis. Soc Sci Med.

[9] Moore S, Bockenholt U, Daniel M, Frohlich K, Kestens Y, Richard L (2011). Social capital and core network ties: a validation study of individual-level social capital measures and their association with extra- and intra-neighborhood ties, and self-rated health. Health Place.

[10] Poortinga W (2006). Social relations or social capital? Individual and community health effects of bonding social capital. Soc Sci Med.

[11] Beaudoin CE (2009). Bonding and bridging neighborliness: an individual-level study in the context of health. Soc Sci Med.

[12] Murayama H, Nishi M, Matsuo E, Nofuji Y, Shimizu Y, Taniguchi Y (2013). Do bonding and bridging social capital affect self-rated health, depressive mood and cognitive decline in older Japanese? A prospective cohort study. Soc Sci Med.

[13] Villalonga-Olives E, Kawachi I (2015). The measurement of bridging social capital in population health research. Health Place.

[14] Clark AK (2015). Rethinking the decline in social capital. Am Politics Res.

[15] Bovens M, Dekker P, Tiemeijer W (2014). Gescheiden werelden.

[16] Li YJ, Savage M, Pickles A (2003). Social capital and social exclusion in England and Wales (1972-1999). Brit J Sociol.

[17] Hvd W (2015). Een kloof van alle tijden. Verschillen tussen lager en hoger opgeleiden in werk, cultuur en politiek.

[18] Oude Groeniger J, van Lenthe FJ, Beenackers MA, Kamphuis CB (2017). Does social distinction contribute to socioeconomic inequalities in diet: the case of 'superfoods' consumption. Int J Behav Nutr Phys Act.

[19] Oude Groeniger J, Kamphuis CB, Mackenbach JP, van Lenthe FJ (2017). Repeatedly measured material and behavioral factors changed the explanation of socioeconomic inequalities in all-cause mortality. J Clin Epidemiol.

[20] Oude Groeniger J, Kamphuis CBM, Mackenbach JP, Beenackers MA, van Lenthe FJ (2019). Are socio-economic inequalities in diet and physical activity a matter of social distinction? A cross-sectional study. Int J Public Health.

[21] Kamphuis CB, Van Lenthe FJ, Giskes K, Huisman M, Brug J, Mackenbach JP (2008). Socioeconomic status, environmental and individual factors, and sports participation. Med Sci Sports Exerc.

[22] Kamphuis CB, Turrell G, Giskes K, Mackenbach JP, van Lenthe FJ (2012). Socioeconomic inequalities in cardiovascular mortality and the role of childhood socioeconomic conditions and adulthood risk factors: a prospective cohort study with 17-years of follow up. BMC Public Health.

[23] Kamphuis CB, Jansen T, Mackenbach JP, van Lenthe FJ (2015). Bourdieu's cultural Capital in Relation to food choices: a systematic review of cultural capital indicators and an empirical proof of concept. PLoS One.

[24] van Wijk DC, Groeniger JO, van Lenthe FJ, Kamphuis CB (2017). The role of the built environment in explaining educational inequalities in walking and cycling among adults in the Netherlands. Int J Health Geogr.

[25] Beenackers Mariëlle A, Oude Groeniger Joost, van Lenthe Frank J, Kamphuis Carlijn B M (2017). The role of financial strain and self-control in explaining health behaviours: the GLOBE study. European Journal of Public Health.

[26] van Lenthe FJ, Kamphuis CB, Beenackers MA, Jansen T, Looman CW, Nusselder WJ (2014). Cohort profile: understanding socioeconomic inequalities in health and health behaviours: the GLOBE study. Int J Epidemiol.

[27] Mackenbach JP, van de Mheen H, Stronks K (1994). A prospective cohort study investigating the explanation of socio-economic inequalities in health in the Netherlands. Soc Sci Med.

[28] Bovens M, Anchrit W (2010). Diplomademocratie. Over de spanning tussen meritocratie en democratie.

[29] Wendel-Vos GC, Schuit AJ, Saris WH, Kromhout D (2003). Reproducibility and relative validity of the short questionnaire to assess health-enhancing physical activity. J Clin Epidemiol.

[30] Bogers RP, Van Assema P, Kester AD, Westerterp KR, Dagnelie PC (2004). Reproducibility, validity, and responsiveness to change of a short questionnaire for measuring fruit and vegetable intake. Am J Epidemiol.

[31] Abel T (2008). Cultural capital and social inequality in health. J Epidemiol Community Health.

[32] Knol MJ, VanderWeele TJ (2012). Recommendations for presenting analyses of effect modification and interaction. Int J Epidemiol.

[33] Ueshima K, Fujiwara T, Takao S, Suzuki E, Iwase T, Doi H (2010). Does social capital promote physical activity? A population-based study in Japan. PLoS One.

[34] Poortinga W (2012). Community resilience and health: the role of bonding, bridging, and linking aspects of social capital. Health Place.

[35] Dammann KW, Smith C (2009). Factors affecting low-income women's food choices and the perceived impact of dietary intake and socioeconomic status on their health and weight. J Nutr Educ Behav.

[36] Gough B, Conner MT (2006). Barriers to healthy eating amongst men: a qualitative analysis. Soc Sci Med.

[37] Mollenhorst G, Volker B, Flap H (2014). Changes in personal relationships: how social contexts affect the emergence and discontinuation of relationships. Soc Networks.

[38] Kandel DB (1978). Similarity in real-life adolescent friendship pairs. J Pers Soc Psychol.

[39] Miller N, Campbell DT, Twedt H, Oconnell EJ (1966). Similarity Contrast and Complementarity in Friendship Choice. J Pers Soc Psychol.

