# Supplementary material for: How does bridging social capital relate to health-behavior, overweight and obesity among low and high educated groups? A cross-sectional analysis of GLOBE-2014
Source: BMC Public Health. 2019 Dec 4;19:1635. doi: 10.1186/s12889-019-8007-3 (PMC6894329; doi:10.1186/s12889-019-8007-3)
Supplement: Supplementary file 1 — Additional file 1. Main effects of educational level and education-specific bridging social capital, adjusted for confounders [file 12889_2019_8007_MOESM1_ESM.docx]

**Additional file 1. Main effects of educational level and education-specific bridging social capital, adjusted for confounders**

**Table A.** Multivariable logistic regression models with odds ratios (OR) and 95% confidence intervals (CI) for health-behavior outcomes, by educational level and bridging social capital, adjusted for confounders^a^ (GLOBE-study, 2014, Eindhoven, The Netherlands).

|  | Daily smoking | | No sports participation | | No leisure time walking | | No leisure time cycling | | No recommended vegetable intake | |
| --- | --- | --- | --- | --- | --- | --- | --- | --- | --- | --- |
|  | ORb | 95% CI | ORb | 95% CI | ORb | 95% CI | ORb | 95% CI | ORb | 95% CI |
| Education |  |  |  |  |  |  |  |  |  |  |
| High | 1.00 |  | 1.00 |  | 1.00 |  | 1.00 |  | 1.00 |  |
| Medium | **2.77***** | **(2.06-3.73)** | **1.47***** | **(1.17-1.82)** | 1.13 | (0.78-1.52) | 1.12 | (0.91-1.38) | **1.74***** | **(1.35-2.24)** |
| Low | **4.61***** | **(3.28-6.48)** | **2.12***** | **(1.65-2.72)** | 1.43 | (1.00-2.04 | **1.31*** | **(1.02-1.69)** | **1.60***** | **(1.20-2.13)** |
| Bridging social capital |  |  |  |  |  |  |  |  |  |  |
| Bonding | 1.00 |  | 1.00 |  | 1.00 |  | 1.00 |  | 1.00 |  |
| Bridging | **1.37**** | **(1.08-1.73)** | **1.25*** | **(1.04-1.51)** | 1.13 | (0.85-1.49) | **1.21*** | **(1.01-1.45)** | **1.39**** | **(1.12-1.72)** |
|  |  |  |  |  |  |  |  |  |  |  |
|  | No recommended fruit intake | | No daily water intake | | High meat intake | | Overweight | | Obesity | |
|  | ORb | 95% CI | ORb | 95% CI | ORb | 95% CI | ORb | 95% CI | ORb | 95% CI |
| Education |  |  |  |  |  |  |  |  |  |  |
| High | 1.00 |  | 1.00 |  | 1.00 |  | 1.00 |  | 1.00 |  |
| Medium | **1.36**** | **(1.09-1.70)** | **1.43**** | **(1.13-1.81)** | 1.21 | (1.02-1.51) | **1.73***** | **(1.40-2.12)** | **1.94***** | **(1.43-2.62)** |
| Low | 1.24 | (0.97-1.61) | **1.53**** | **(1.17-2.01)** | **1.46**** | **(1.15-1.86)** | **1.90***** | **(1.49-2.42)** | **2.02***** | **(1.45-2.82)** |
| Bridging social capital |  |  |  |  |  |  |  |  |  |  |
| Bonding | 1.00 |  | 1.00 |  | 1.00 |  | 1.00 |  | 1.00 |  |
| Bridging | 1.07 | (0.89-1.29) | 0.99 | (0.81-1.21) | 1.06 | (0.89-1.26) | 0.95 | (0.79-1.13) | 0.87 | (0.68-1.12) |

a. For each health-behavior outcome, we run a separate model including the following variables: educational level, bridging social capital, sex, age, employment status, country of birth, living with a partner, living with children in household, father’s educational level, and mother’s educational level.

b. * = p < .050, ** = p < .010, *** = p < .001
