# Supplementary material for: How does bridging social capital relate to health-behavior, overweight and obesity among low and high educated groups? A cross-sectional analysis of GLOBE-2014
Source: BMC Public Health. 2019 Dec 4;19:1635. doi: 10.1186/s12889-019-8007-3 (PMC6894329; doi:10.1186/s12889-019-8007-3)
Supplement: Supplementary file 2 — Additional file 2. Models for associations between income-specific bridging social capital and health-behavior, modified by income level [file 12889_2019_8007_MOESM2_ESM.docx]

**Additional file 2: Models for associations between income-specific bridging social capital and health-behavior, modified by income level**

**Table B1.** Multivariable logistic regression models for modification of the association between income-specific bridging social capital^a^ and daily smoking, no sports participation, no leisure time walking, and no leisure time cycling, by income level (GLOBE-study, 2014, Eindhoven, The Netherlands)

|  | Bonding social capital^a^ | | | Bridging social capital^a^ | | | ORs (95% CI) for bridging capital within income groups | |
| --- | --- | --- | --- | --- | --- | --- | --- | --- |
| Daily smoking | | | |  | | |  | |
| Income | N smoking vs.  N non-smoking | OR ^b^ | (95% CI) | N smoking vs.  N non-smoking | OR^b^ | (95% CI) | OR^c^ | (95% CI) |
| High | 11 vs. 206 | 1.00 |  | 14 vs. 169 | 1.29 | (0.60-2.78) | 1.23 | (0.54-2.84) |
| Mid-high | 31 vs. 337 | 1.22 | (0.62-2.39) | 43 vs. 275 | 1.74 | (0.90-3.39) | 1.41 | (0.87-2.28) |
| Mid-low | 32 vs. 229 | 1.36 | (0.67-2.74) | 49 vs. 247 | 1.75 | (0.89-3.45) | 1.20 | (0.73-1.97) |
| Low | 38 vs. 148 | 1.37 | (0.65-2.90) | 103 vs. 262 | **2.31*** | **(1.18-4.55)** | **1.73** | **(1.09-2.75)** |
| No sports participation | | | |  |  |  |  |  |
| Income | N no sports vs. N doing sports | OR ^b^ | (95% CI) | N no sports vs.  N doing sports | OR^b^ | (95% CI) | OR^c^ | (95% CI) |
| High | 40 vs. 162 | 1.00 |  | 41 vs.132 | 1.27 | (0.80-2.04) | 1.26 | (0.75-2.12) |
| Mid-high | 106 vs. 233 | 1.36 | (0.90-2.04) | 92 vs. 198 | 1.58 | (1.04-2.40) | 1.10 | (0.78-1.56) |
| Mid-low | 102 vs. 148 | 1.81 | (1.17-2.82) | 108 vs. 171 | 1.48 | (0.95-2.29) | 0.82 | (0.56-1.20) |
| Low | 76 vs. 101 | 1.48 | (0.95-2.29) | 168 vs. 171 | **1.94**** | **(1.18-3.21)** | 1.15 | (0.77-1.73) |
| No leisure time walking | | | |  |  |  |  |  |
| Income | N no walking vs. N walking | OR^b^ | (95% CI) | N no walking vs.  N walking | OR^b^ | (95% CI) | OR^c^ | (95% CI) |
| High | 154 vs. 47 | 1.00 |  | 135 vs. 42 | 0.66 | (0.34-1.27) | 0.49 | (0.21-1.12) |
| Mid-high | 251 vs. 105 | 1.05 | (0.59-1.89) | 216 vs. 82 | 1.04 | (0.56-1.91) | 1.04 | (0.60-1.83) |
| Mid-low | 151 vs. 99 | 0.95 | (0.50-1.78) | 172 vs. 103 | 0.97 | (0.52-1.83) | 1.01 | (0.59-1.74) |
| Low | 106 vs. 67 | 0.59 | (0.28-1.22) | 223 vs. 114 | 0.91 | (0.48-1.74) | 1.57 | (0.87-2.82) |
| No leisure time cycling | | | |  |  |  |  |  |
| Income | N no cycling vs.  N cycling | OR^b^ | (95% CI) | N no cycling vs.  N cycling | OR^b^ | (95% CI) | OR^c^ | (95% CI) |
| High | 81 vs. 118 | 1.00 |  | 81 vs. 90 | **1.57*** | **(1.05-2.33)** | **1.60*** | **(1.04-2.46)** |
| Mid-high | 140 vs. 210 | 1.01 | (0.71-1.44) | 133 vs. 161 | 1.23 | (0.86-1.77) | 1.21 | (0.88-1.67) |
| Mid-low | 98 vs. 149 | 1.10 | (0.74-1.63) | 117 vs. 158 | 1.18 | (0.80-1.75) | 1.07 | (0.74-1.57) |
| Low | 70 vs. 106 | 1.00 | (0.63-1.59) | 141 vs. 185 | 1.17 | (0.78-1.76) | 1.14 | (0.77-1.69) |

* = p < .050, ** = p < .010, *** = p < .001

^a^ Income-specific bridging social capital was measured with the question: “How many of your close friends have the same income level as you have?”. Bridging social capital was coded as ‘1=bridging’ for those who answered ‘about half’, ‘some’, or ‘none of my friends’, and ‘0=bonding’ for those who answered ‘all’ or ‘most of my friends’.

^b^ Odds ratios and 95% confidence intervals regressing the outcomes on the ‘income x capital’-variable and confounders (sex, age, employment status, country of birth, living with a partner, children in household, father’s education, mother’s education).

^c^ Odds ratios and 95% confidence intervals regressing the outcomes on bridging social capital and confounders, stratified by income level.
- Measures on multiplicative scale for smoking: OR income mid-high* bridging cap = 1.13 (95% CI 0.46-2.79), P=.784; OR income mid-low* bridging cap = 1.02 (95% CI 0.41-2.54), P=.959; OR income low* bridging cap: = 1.37 (95% CI 0.56-3.35), P=.497.

- Measures on multiplicative scale regarding no sports: : OR income mid-high* bridging cap = 0.92 (95% CI 0.52-1.64), P=.787; OR income mid-low* bridging cap = 0.64 (95% CI 0.35-1.16), P=.143; OR income low* bridging cap = 0.90 (95% CI .0.49-1.65), P=.723.

- Measures on multiplicative scale regarding no walking: : OR income mid-high* bridging cap= 1.50 (95%CI 0.65-3.45), P=.339; OR income mid-low* bridging cap=1.59 (95%CI 0.68-3.73), P=.290; OR income low* bridging cap= **2.50 (95%CI 1.03-6.09),P=.044.**
- Measures on multiplicative scale regarding no cycling: OR income mid-high* bridging cap= 0.77 (95% CI 0.46-1.28), P=.309; OR income mid-low* bridging cap= 0.68 (95% CI 0.40-1.17), P=.165; OR income low* bridging cap= 0.74 (95% CI 0.42-1.29), P=.283.

**Table B2.** Multivariable logistic regression models for modification of the association between income-specific bridging social capital^a^ and food intake, by income level (GLOBE-study, 2014, Eindhoven, The Netherlands)

|  | Bonding social capital^a^ | | | Bridging social capital^a^ | | | ORs (95% CI) for bridging capital within income groups | |
| --- | --- | --- | --- | --- | --- | --- | --- | --- |
| No recommended vegetable intake | | | |  | | |  | |
| Income | N no recomm. vs. N recomm. veg. intake | OR ^b^ | (95% CI) | N no recomm. vs. N recomm. veg. intake | OR^b^ | (95% CI) | OR^c^ | (95% CI) |
| High | 146 vs. 69 | 1.00 |  | 134 vs. 48 | 1.36 | (0.88-2.09) | 1.36 | (0.86-2.15) |
| Mid-high | 277 vs. 87 | 1.42 | (0.98-2.07) | 227 vs. 88 | 1.06 | (0.72-1.54) | 0.72 | (0.51-1.02) |
| Mid-low | 189 vs. 72 | 1.06 | (0.70-1.60) | 241 vs. 51 | **1.86*** | **(1.19-2.88)** | **1.75**** | **(1.14-2.68)** |
| Low | 150 vs. 36 | **1.68*** | **(1.01-2.81)** | 282 vs. 76 | **1.58*** | **(1.01-2.48)** | 0.92 | (0.57-1.48) |
| No recommended fruit intake | | | |  |  |  |  |  |
| Income | N no recomm. vs. N recomm. fruit intake | OR ^b^ | (95% CI) | N no recomm. vs. N recomm. fruit intake | OR^b^ | (95% CI) | OR^c^ | (95% CI) |
| High | 138 vs. 79 | 1.00 |  | 122 vs. 61 | 1.32 | (0.88-1.98) | 1.33 | (0.87-2.05) |
| Mid-high | 228 vs. 140 | 1.18 | (0.84-1.67) | 218 vs. 97 | 1.39 | (0.97-2.00) | 1.15 | (0.83-1.60) |
| Mid-low | 164 vs. 99 | 1.27 | (0.86-1.88) | 193 vs. 101 | 1.28 | (0.87-1.89) | 1.01 | (0.70-1.46) |
| Low | 122 vs. 64q | **1.65*** | **(1.03-2.63)** | 260 vs.97 | **1.77**** | **(1.17-2.68)** | 1.08 | (0.71-1.64) |
| No daily water | | | |  |  |  |  |  |
| Income | N no daily water vs. N daily water intake | OR^b^ | (95% CI) | N no daily water vs. N daily water intake | OR^b^ | (95% CI) | OR^c^ | (95% CI) |
| High | 42 vs. 175 | 1.00 |  | 31 vs. 151 | 0.85 | (0.52-1.38) | 0.73 | (0.43-1.24) |
| Mid-high | 76 vs. 291 | 1.11 | (0.73-1.68) | 70 vs. 246 | 1.17 | (0.77-1.78) | 1.00 | (0.69-1.44) |
| Mid-low | 69 vs. 193 | 1.64 | (1.05-2.56) | 70 vs. 225 | 1.25 | (0.80-1.95) | 0.74 | (0.50-1.11) |
| Low | 40 vs. 149 | 1.44 | (0.85-2.44) | 92 vs. 273 | 1.42 | (0.89-2.25) | 1.04 | (0.68-1.60) |
| High meat intake | | | |  |  |  |  |  |
| Income | N high meat vs.  N no high meat intake | OR^b^ | (95% CI) | N high meat vs.  N no high meat intake | OR^b^ | (95% CI) | OR^c^ | (95% CI) |
| High | 130 vs. 87 | 1.00 |  | 106 vs. 76 | 0.92 | (0.62-1.36) | 0.80 | (0.53-1.21) |
| Mid-high | 223 vs. 144 | 1.00 | (0.71-1.40) | 187 vs. 130 | 0.95 | (0.67-1.34) | 0.97 | (0.72-1.31) |
| Mid-low | 148 vs. 116 | 0.92 | (0.63-1.34) | 189 vs. 107 | 1.21 | (0.83-1.77) | 1.28 | (0.89-1.84) |
| Low | 103 vs. 86 | 1.06 | (0.68-1.65) | 175 vs. 191 | 0.80 | (0.54-1.17) | 0.71 | (0.49-1.02) |

* = p < .050, ** = p < .010, *** = p < .001

^a^ Income-specific bridging social capital was measured with the question: “How many of your close friends have the same income level as you have?”. Bridging social capital was coded as ‘1=bridging’ for those who answered ‘about half’, ‘some’, or ‘none of my friends’, and ‘0=bonding’ for those who answered ‘all’ or ‘most of my friends’.

^b^ Odds ratios and 95% confidence intervals regressing the outcomes on the ‘income x capital’-variable and confounders (sex, age, employment status, country of birth, living with a partner, children in household, father’s education, mother’s education).

^c^ Odds ratios and 95% confidence intervals regressing the outcomes on bridging social capital and confounders, stratified by income level.
 - Measures on multiplicative scale for no vegetables: OR income mid-high* bridging cap = 0.55(95% CI 0.32-0.95), P=.033.; OR income mid-low* bridging cap = 1.30 (95% CI0.71-2.36), P=.394; OR income low* bridging cap= 0.70 (95% CI0.38-1.32), P=.270.

- Measures on multiplicative scale regarding no fruits: OR income mid-high* bridging cap = 0.90 (95% CI 0.54-1.50), P=.677; OR income mid-low* bridging cap = 0.76(95% CI 0.44-1.30), P=.314; OR income low* bridging cap = 0.83 (95% CI 0.47-1.46), P=.510

- Measures on multiplicative scale regarding no daily water: OR income mid-high* bridging cap= 1.27 (95%CI 0.70-2.31), P=.440; OR income mid-low* bridging cap= 0.91 (95%CI 0.49-1.69) P=.773; OR income low* bridging cap= 1.18 (95%CI 0.62-2.24),P=.620
- Measures on multiplicative scale regarding high meat: OR income mid-high* bridging cap= 1.04 (95% CI 0.64-1.69), P=.882; OR income mid-low* bridging cap= 1.42(95% CI0.84-2.38), P=.189; OR income low* bridging cap= 0.80 (95% CI0.47-1.36), P=.411.

**Table B3.** Multivariable logistic regression models for modification of the association between income-specific bridging social capital^a^ and overweight and obesity, by income level (GLOBE-study, 2014, Eindhoven, The Netherlands)

|  | Bonding social capital^a^ | | | Bridging social capital^a^ | | | ORs (95% CI) for bridging capital within income groups | |
| --- | --- | --- | --- | --- | --- | --- | --- | --- |
| Overweight | | | |  | | |  | |
| Income | N overweight vs. N no overweight | OR ^b^ | (95% CI) | N overweight vs. N no overweight | OR^b^ | (95% CI) | OR^c^ | (95% CI) |
| High | 73 vs. 144 | 1.00 |  | 68 vs. 114 | 1.12 | (0.74-1.69) | 1.04 | (0.66-1.63) |
| Mid-high | 144 vs. 221 | 1.27 | (0.89-1.81) | 141 vs. 173 | **1.49*** | **(1.03-2.15)** | 1.17 | (0.85-1.59) |
| Mid-low | 119 vs. 139 | **1.51*** | **(1.01-2.26)** | 168 vs. 125 | **2.18***** | **(1.47-3.22)** | **1.48*** | **(1.02-2.16)** |
| Low | 100 vs. 89 | **1.92**** | **(1.21-3.05)** | 175 vs. 184 | **1.70*** | **(1.13-2.55)** | 0.92 | (0.61-1.38) |
| Obesity | | | |  |  |  |  |  |
| Income | N obesity vs.  N no obesity | OR ^b^ | (95% CI) | N obesity vs.  N no obesity | OR^b^ | (95% CI) | OR^c^ | (95% CI) |
| High | 10 vs. 207 | 1.00 |  | 12 vs. 170 | 1.92 | (0.79-4.68) | 1.05 | (0.35-3.11) |
| Mid-high | 36 vs. 329 | **2.56*** | **(1.17-5.59)** | 46 vs.268 | **3.38**** | **(1.70-8.39)** | 1.25 | (0.78-1.98) |
| Mid-low | 43 vs. 215 | **3.78***** | **(1.70-8.39)** | 54 vs. 239 | **3.84***** | **(1.74-8.44)** | 1.19 | (0.74-1.94) |
| Low | 32 vs. 157 | **3.24**** | **(1.37-7.68)** | 66 vs. 293 | **3.57**** | **(1.60-7.99)** | 1.11 | (0.66-1.87) |

* = p < .050, ** = p < .010, *** = p < .001

^a^ Income specific bridging social capital was measured with the question: “How many of your close friends have the same income level as you have?”. Bridging social capital was coded as ‘1=bridging’ for those who answered ‘about half’, ‘some’, or ‘none of my friends’, and ‘0=bonding’ for those who answered ‘all’ or ‘most of my friends’.

^b^ Odds ratios and 95% confidence intervals regressing the outcomes on the ‘income x capital’-variable and confounders (sex, age, employment status, country of birth, living with a partner, children in household, father’s education, mother’s education).

^c^ Odds ratios and 95% confidence intervals regressing the outcomes on bridging social capital and confounders, stratified by income level
- Measures on multiplicative scale regarding overweight: OR income mid-high* bridging cap= 1.05 (95%CI 0.63-1.76), P=.846; OR income mid-low* bridging cap= 1.26 (95%CI 0.73-2.18) P=.406; OR income low* bridging cap= 0.76 (95%CI 0.43-1.34),P=.350.
- Measures on multiplicative scale regarding obesity: OR income mid-high* bridging cap= 0.68 (95% CI 0.25-1.85), P=.682; OR income mid-low* bridging cap= 0.51 (95% CI 0.19-1.39), P=.190; OR income low* bridging cap= 0.57 (95% CI 0.21-1.58), P=.279
